# Irritable Bowel Syndrome Care in Catalonia: A Qualitative Study with Healthcare Professionals on the Management of Uncertainty and the Possibilities of Technological and Digital Developments

**DOI:** 10.3390/healthcare14182928

**Published:** 2026-09-09

**Authors:** Eduard Moreno Gabriel, Faranak Nooriankafshgari, Rosa García-Sierra, Candela Sancho Vallvé, Daina Parellada-Moreno, Victoria Ardiles Ruesjas, Immaculada Herrero-Fresneda, Pere Torán-Monserrat

**Affiliations:** 1 Unitat de Suport a la Recerca (USR) Metropolitana Nord, Fundació Institut Universitari d’Investigació en Atenció Primària Jordi Gol i Gurina (IDIAP Jordi Gol), 08303 Mataró, Spain; emorenoga.mn.ics@gencat.cat (E.M.G.); rgarciasi.mn.ics@gencat.cat (R.G.-S.); csanchova.mn.ics@gencat.cat (C.S.V.); dparellada@idiapjgol.org (D.P.-M.); vardiles@idiapjgol.org (V.A.R.); ptoran.bnm.ics@gencat.cat (P.T.-M.); 2 Grup de Recerca Multidisciplinar en Salut i Societat (GREMSAS) 2021 SGR 01484, IDIAP Jordi Gol, USR MN, 08303 Mataró, Spain; 3 Nursing Department, Faculty of Medicine, Universitat Autònoma de Barcelona, 08193 Barcelona, Spain; 4 USMIMA S.L, Av. Cornellà 140 6A, Esplugues de Llobregat, 08950 Barcelona, Spain; ihf@mowoot.com; 5 Department of Medical Sciences, Faculty of Medicine, Universitat de Girona, 17003 Girona, Spain

**Keywords:** irritable bowel syndrome, primary healthcare, qualitative research, clinical uncertainty, digital health, patient management, gut–brain interaction, non-pharmacological intervention

## Abstract

**Highlights:**

**What are the main findings?**

- Primary care management of irritable bowel syndrome functions as a liminal hotspot characterized by the navigation of persistent clinical uncertainty, fluctuating symptoms, and brief, repeated consultations.
- Participating clinicians reported relying on a foundation of exclusion and longitudinal pattern recognition, utilizing relational knowledge and patient narratives rather than definitive biomarkers.

**What are the implications of the main findings?**

- Comprehensive care requires a biopsychosocial approach that integrates lifestyle adjustments, patient education, and therapeutic communication alongside clinical guidelines.
- Digital and support tools should prioritize low professional burden and clinically interpretable data to organize symptom trajectories and strengthen the therapeutic alliance.

**Abstract:**

Background/Objectives: Irritable bowel syndrome (IBS) is one of the most common disorders of gut–brain interaction and one of the most frequent functional bowel disorders encountered in clinical practice. The lack of reliable biomarkers, coupled with inconsistent clinical presentations and variable therapeutic outcomes, creates a landscape of ongoing uncertainty for clinicians. Against this background, non-pharmacological and technological developments—such as digital health interfaces, sensor technologies, and medical devices—emerge as potential support tools for clinical management. This qualitative study explored how healthcare professionals manage diagnostic and therapeutic uncertainty in IBS across care settings while evaluating the potential role and value of integrating these technological and non-pharmacological innovations into routine practice. Methods: Fifteen semi-structured interviews were conducted with healthcare professionals involved in IBS care across primary and specialized settings and one expert patient with lived experience in the Barcelona metropolitan area. Data were analyzed using principles of grounded theory. Results: The notion of “catch-all category” articulates four interrelated themes that characterize IBS in clinical practice: persistent clinical uncertainty, pragmatic diagnostic strategies, patient engagement in biopsychosocial and longitudinal management, and the implementation and interpretability of digital and non-pharmacological support. Participants described IBS management as shifting from exclusionary, symptom-based diagnostics toward holistic biopsychosocial and gut–brain-axis models. This transition underscores the necessity for resources that empower patients through education, support clinicians through longitudinal monitoring, and facilitate shared management strategies. Conclusions: The findings suggest that IBS management is not a single diagnostic act but a longitudinal process of uncertainty management. Future digital or non-pharmacological support tools for IBS should prioritize longitudinal monitoring, clinically interpretable information, and low burden for professionals and patient–professional communication.

## 1. Introduction

Irritable bowel syndrome (IBS) is one of the most common disorders of gut–brain interaction and one of the most frequent functional bowel disorders encountered in clinical practice. It is characterized by recurrent abdominal pain, bloating or abdominal distension, and altered bowel habits, with constipation, diarrhea, or mixed bowel patterns, in the absence of structural or biochemical abnormalities that explain the symptoms [1,2,3,4,5,6]. Current prevalence estimates vary depending on the diagnostic criteria used. A global systematic review and meta-analysis reported a pooled prevalence of 9.2% using Rome III criteria and 3.8% using Rome IV criteria [7]. Similarly, the Rome Foundation Global Epidemiology Study showed that disorders of gut–brain interaction are highly prevalent worldwide and are associated with substantial healthcare burden and impaired quality of life [8]. Clinical reviews commonly estimate that IBS affects approximately 5–10% of the general population at any given time [6].

Beyond its prevalence, IBS has a substantial impact on patients’ daily lives and consequently on healthcare systems. Symptoms often follow a relapsing-remitting course and may interfere with social activities, work, diet, emotional wellbeing, and quality of life. The socioeconomic burden includes both direct healthcare costs, such as repeated consultations, diagnostic testing and treatments, and indirect costs related to absenteeism and reduced productivity [6,9,10]. IBS is particularly relevant for primary care because many patients are diagnosed, followed, and managed outside specialist gastroenterology. Earlier Spanish clinical guidance estimated that IBS accounted for approximately 3% of primary care consultations and 16–25% of gastroenterology consultations [9]. Although these figures should be interpreted cautiously because they come from older studies, they support the view that IBS is among the most frequent digestive reasons for consultation in primary care and gastroenterology. This primary care relevance is not only epidemiological but also organizational: patients often require repeated consultations, diagnostic reassurance, selective exclusion of organic disease, and longitudinal management of symptoms over time [6,10,11].

Even though IBS is highly prevalent and frequently managed outside specialist gastroenterology, it remains clinically challenging because symptoms are heterogeneous, fluctuate over time, and are influenced by dietary, psychosocial, and physiological factors [1,2,3,4,5]. In routine primary care, its management requires clinicians to integrate symptom interpretation, exclusion of alarm features, patient education and reassurance, and symptom-guided treatment decisions within short, often repeated consultations [12,13,14,15,16]. This complexity underscores that IBS is defined not only by its diagnostic difficulty but also by the need for sustained, longitudinal management of uncertainty. In practice, management is often pragmatic: while formal diagnostic criteria such as Rome IV are important, they may not be fully practicable in everyday consultations, where time constraints require clinicians to balance diagnostic evaluation, patient reassurance, and therapeutic interventions [12,13,14,15]. The absence of a single definitive biomarker reinforces a form of diagnostic strategy that relies on longitudinal knowledge of the patient, interpretation of symptom narratives, and assessment of response to lifestyle, dietary, pharmacological, or psychological interventions. This diagnostic uncertainty extends directly to treatment. Therapeutic approaches are also similarly uncertain and typically focus on the most troublesome symptoms, including constipation, diarrhea, bloating, and pain. However, these symptom-based strategies do not always provide lasting benefits, and patients may undergo a prolonged process of trial and error. This is particularly relevant in primary care, where IBS is managed over repeated encounters rather than through a single specialist episode [7,10,16,17]. Against this background of diagnostic and therapeutic uncertainty, new approaches to IBS management are being developed. Digital health tools combined with non-pharmacological interventions have been proposed as complementary strategies for IBS management, particularly when they support self-management, symptom monitoring, patient education, and brain–gut behavioral approaches [18,19]. A recent systematic review of digital health interventions for IBS identified education, diet, brain–gut behavior skills, physiological support, health monitoring, and community engagement as key intervention domains, with evidence suggesting improvements in IBS symptoms and quality of life, although feasibility and generalizability remain uncertain [19]. In parallel, smartphone-based symptom diaries have been shown to support the standardized, time-stamped collection of patient-reported outcomes and to capture symptom variability over time [20].

The MOWOOT-IBS project provides the context for the present study. It seeks to develop and evaluate a non-invasive intervention that integrates a medical device with digital support tailored to irritable bowel syndrome, particularly the constipation-predominant subtype (IBS-C). However, implementation research in digital health shows that adoption depends not only on clinical promise but also on usability, perceived value, organizational context, and integration with professional workflows. Technologies that increase workload, generate poorly actionable information, or fail to fit into routine care may face non-adoption or abandonment [21]. Therefore, understanding how clinicians manage IBS-related uncertainty, what information they consider clinically useful, and what types of support they value in routine practice is essential to inform the development of MOWOOT-IBS and similar digital or non-pharmacological interventions.

While previous qualitative research on irritable bowel syndrome (IBS) has primarily focused on the patient’s lived experience or explored general practitioner attitudes in isolation, significant knowledge gaps remain regarding how uncertainty is navigated across multidisciplinary teams in everyday practice. Specifically, little is known about how clinicians across different levels of care—primary care physicians, nurses, gastroenterologists, and rehabilitation specialists—collaboratively manage the lack of definitive biomarkers while integrating digital and non-pharmacological tools into routine workflows. Existing models often portray diagnosis as a discrete decision-making event; however, it remains unclear how multidisciplinary teams create practical management strategies when consultation time is severely limited.

Although IBS has been widely studied from biomedical, epidemiological, and therapeutic perspectives, less is known about how professionals make sense of IBS-related uncertainty in everyday consultations, what information they consider clinically useful, and how these needs should inform the design of digital or non-pharmacological support tools. Therefore, the present study aims to explore how healthcare professionals manage diagnostic and therapeutic uncertainty associated with IBS in real-world clinical settings in the Barcelona metropolitan area, across both primary and specialized care settings. It also seeks to identify the types of information clinicians consider most useful and to examine how these needs may inform the development of digital and non-pharmacological support tools [22,23].

## 2. Materials and Methods

### 2.1. Study Design

This study adopted an exploratory qualitative design within a constructivist research paradigm, which is appropriate for investigating complex phenomena and subjective perceptions in clinical environments. A constructivist approach assumes that reality is socially constructed and shaped through individuals’ experiences and interactions, making it suitable for exploring how healthcare professionals understand and manage diagnostic and therapeutic uncertainty. In addition, no single formal guiding theory underpinned the study; thus, this approach follows the precepts of Charmaz’s constructivist version of Grounded Theory [24] to develop an in-depth exploration of participants’ perspectives and provide a coherent account of the variability of clinical decision-making processes.

This qualitative inquiry was embedded within the broader context of the MOWOOT-IBS project, an initiative evaluating a non-invasive medical device and digital support ecosystem for IBS-C. To prevent response bias and ensure data integrity, the MOWOOT device was not introduced as a primary focal point during interviews. Instead, the study design employed open-ended framing to explore clinicians’ general management of diagnostic and therapeutic uncertainty, their overall perceptions of digital health, and the practical usability of non-pharmacological tools in routine workflows. This allowed participants to freely voice both criticism and enthusiasm regarding technology integration without being nudged toward a specific commercial intervention.

The study was conducted in the Barcelona metropolitan area across primary and specialized care settings. This context was selected to capture diverse clinical perspectives within the Spanish public healthcare system, where coordination between care levels shapes the management of diagnostic and therapeutic uncertainty in IBS.

### 2.2. Setting and Participants

The sampling was intentional and theoretically oriented, designed to ensure diversity according to the following criteria: (a) professional experience contrasting junior and senior profiles, (b) representation of different levels of care—primary and specialized care, (c) variability in clinical disciplines, (d) profiles with clinical care experience and/or academic/research trajectories, and (e) incorporation of a patient’s perspective to guide methodological choices. While the primary units of study were healthcare professionals (focusing on their experiences and perspectives on managing diagnostic and therapeutic uncertainty in IBS), one expert patient with lived experience of IBS was included as a key informant and pilot participant to refine the research tools and provide foundational lived experience context.

Participants were initially identified through the project’s clinical research team, and recruitment was subsequently expanded using a snowball strategy, through which participants recommended additional key informants to ensure access to relevant perspectives. A total of 15 interviews were conducted (N = 15), including 14 healthcare professionals across primary and specialized care settings and 1 expert patient with lived experience of IBS who provided exploratory pilot feedback (see Table 1).

After conducting 12 interviews, we observed data redundancy indicative of data saturation. Nevertheless, we continued data collection to incorporate diverse clinical approaches to IBS management (e.g., from a rehabilitation specialist). Although these participants introduced novel research avenues (such as exploring alternative explanatory frameworks for IBS in the absence of a clear-cut diagnosis), their accounts of clinical practice were fully aligned with our emerging theoretical framework, reinforcing its theoretical sufficiency [25].

### 2.3. Data Collection

Data were collected through semi-structured individual interviews, which were suitable for an in-depth exploration of participants’ perspectives. Interviews were conducted by the first author, E.M.G., a social psychologist trained in qualitative methods, without previous experience or professional knowledge of IBS, but institutionally related to some of the participants as a member of the research unit of their primary healthcare organization. The interviews were conducted both in person and remotely between April and December 2024. They had an average duration of thirty minutes and were audio-recorded, transcribed verbatim, and anonymized. Field notes and analytical memos were generated during the process. No repeat interviews were conducted; all 15 participants completed a single semi-structured interview session.

A script of open-ended questions (see Supplementary Materials File S1) was prepared and tested in consultation with an expert patient with lived experience of IBS during the early protocol design stage. Following the approval of the ethical amendment (CEI IDIAP Jordi Gol code 24/097-P), the expert patient was formally interviewed to provide further details on living with IBS and care pathways. This transcript served a dual purpose: providing direct patient context on care pathways and functioning as a key informant source included in the overall qualitative analysis alongside the healthcare professional interviews. As a result, subsequent interviews with healthcare professionals explored the following themes: the diagnostic process, clinical signs and symptoms considered relevant, use of tests, treatment options, barriers and facilitators in routine care, perceptions of the gut–brain axis, and potential roles for technological or non-pharmacological support.

### 2.4. Data Analysis

Data analysis followed procedures informed by constructivist Grounded Theory [24], involving an iterative process between data collection and analysis. Focused coding enabled grouping these codes into broader analytical categories, which constituted the four main themes characterizing IBS management: the paradox of persistent uncertainty, pragmatic diagnostic strategies, the transition from symptom control to biopsychosocial and longitudinal management, and the potential of digital and non-pharmacological support. These dynamics were theoretically integrated around the core category of the “catch-all category”. This overarching concept articulates how IBS is operationalized in clinical practice—functioning as both a rhetorical and practical framework to navigate and manage persistent clinical uncertainty.

Analytical memos were used to serve several purposes throughout the study. Mainly, these provided a space for making comparisons across participant groups and emerging codes and categories. These analytical memos, alongside team debriefings, were used to support reflexivity, facilitate triangulation within the research team, and enhance analytical rigor by identifying deviant cases from emerging theorizations.

Because this study was conducted alongside a technology development initiative, the research team applied continuous reflexivity during data collection, coding, and interpretation to mitigate confirmation bias. Participants were informed of this feature towards the end of the interviews. The author affiliated with USMIMA endorsed this exploratory project and gained access to relevant insights but was not involved in any of the fieldwork procedures (recruitment, data collection, analysis, and interpretation). The program ATLAS.ti (version 24, ATLAS.ti Scientific Software Development GmbH, Berlin, Germany) was used to facilitate data organization and coding.

Illustrative quotations were selected to represent the main themes identified (see Section 3). Quotations originally provided in Catalan or Spanish were translated into English by the authors and checked against the original transcripts to preserve participants’ intended meaning. Where necessary, quotations were lightly edited for readability, and summarized quotations were identified in the text.

### 2.5. Ethical Considerations

The study involved human participants and was conducted in accordance with the Declaration of Helsinki and the rules of Good Clinical Practice (GCP). The processing of personal data is carried out in accordance with the General Data Protection Regulation (GDPR, EU 2016/679) and the applicable national legislation. The study protocol was approved by the Clinical Research Ethics Committee of IDIAP Jordi Gol (CEIm IDIAP Jordi Gol [24/097-P]). An ethical amendment explicitly covering the formal interview and inclusion of the expert patient as a key informant in the qualitative analysis was subsequently approved on 14 October 2024. All participants gave informed consent before study involvement. Pseudonymization of information was guaranteed to preserve the confidentiality and anonymity of participants in all phases of analysis and dissemination of results.

The research team was composed of health researchers with experience in both clinical contexts and qualitative methods; reflexive awareness was maintained throughout the study to consider how prior assumptions and professional backgrounds may have influenced data collection, analysis, and interpretation. The study is reported according to the GUREGT guideline for reporting Grounded Theory studies [26].

## 3. Results

### 3.1. IBS as a Catch-All Category

One general practitioner emphasized that IBS is considered a syndrome rather than a specific disease. More precisely, she described it as a “tailor’s drawer,” a Catalan expression meaning a “catch-all category,” used to refer to a heterogeneous set of symptoms that are not fully explained by other conditions.

*“I: […], we’re doing interviews about… Irritable bowels or irritable colon or…? How do you call it?*

*P1: Generally, it’s irritable bowel syndrome, because it is not a specific disease in itself, but rather it is a bit… I consider it a catch-all category, a collection of many symptoms that are not explained by anything else.*

*I: What do you mean by a “tailor drawer”?*

*P1: It means that to diagnose irritable bowel syndrome you rule out many other intestinal or stomach diseases. Then, in the end, when everything comes back negative and there is nothing else and it fits certain minimum criteria for diarrhea, bloating, discomfort, then it ends up being irritable bowel syndrome.”*

(General Practitioner, EE03:1)

As this participant noted, these symptoms could overlap with other gastrointestinal problems; therefore, classifying this collection of symptoms as IBS is often the “end” point of a process in which diagnosis operates through a logic grounded in diagnostic exclusion rather than discovery, serving instead as a way of organizing clinical knowledge and practices. The term ‘IBS’ is a space where several heterogeneous elements are assembled and also a set of rather disorganized epistemic and practical resources that help professionals navigate their relationships with patients.

Despite this structural ambiguity, our participants describe IBS management by way of four interrelated characteristics: persistent uncertainty, pragmatic diagnostic strategies, patient engagement, and potential/promising treatments.

These findings describe IBS management across four empirical themes: the paradox of persistent uncertainty, pragmatic diagnostic strategies, from symptom control to biopsychosocial and longitudinal management, and the potential of digital and non-pharmacological support.

The perception of IBS as a ‘catch-all category’ varied substantially across professional roles and care settings. Primary care physicians frequently described IBS as an operational ‘catch-all’ or residual diagnostic umbrella, driven by tight consultation time limits and limited access to specialized diagnostic testing (e.g., breath tests for fructose). For primary care physicians, the category served as a pragmatic default when alarm features were absent, though several expressed underlying concern that unmeasured organic pathologies remained undetected.

In contrast, specialized gastroenterologists contested the ‘catch-all’ characterization. Specialists emphasized that when diagnostic criteria (such as Rome IV) are applied rigorously, IBS represents a distinct functional entity rather than a dumping ground for unexplained abdominal symptoms. However, gastroenterologists acknowledged that in primary care practice, the label is often applied prematurely as a diagnosis of exclusion. Nurses and rehabilitation specialists viewed the concept through a management lens, focusing less on the diagnostic label itself and more on the functional impact of persistent symptoms regardless of the underlying nomenclature.

### 3.2. The Paradox of Persistent Uncertainty

Participants described IBS as a condition in which uncertainty was not an exceptional event but a defining and persistent feature of routine IBS management.

As the primary care physician explains below, rather than a given episode or symptom, it is a pattern of repeated consultations and symptoms that contribute to building diagnostic suspicion around IBS:

*“P9: A short episode of diarrhea alone does not guide you, but when the patient comes repeatedly with diarrhea, abdominal discomfort or altered bowel transit, then you begin to suspect that it may be related to irritable bowel syndrome. First, you rule out the worst possibilities.”*

(Primary care physician, EE09)

Although repeated consultations help clinicians build diagnostic confidence, this pattern must be paradoxically uncertain to guide professionals towards the IBS diagnosis. Often, symptoms are not only chronic but also fluctuating. Professionals emphasized that pain, constipation, diarrhea, bloating, stress, diet, and daily functioning may interact differently across patients. Consequently, clinical confidence emerges gradually from the interaction between repeated observation and “confirmed uncertainty”. That is, professionals describe IBS not only as lacking a definitive symptom but also as a condition without a definitive confirmatory test. This repeated cycle transforms the primary care consultation into a “liminal hotspot”—a site of ongoing transition where clinician and patient remain stuck between ruling out organic disease and managing persistent, non-specific symptoms. One primary care physician described the diagnostic process as a necessary “road to nowhere” where IBS emerges in the confirmed absence of certainty regarding other pathologies:

*“P4: Once we have done the whole battery of tests and do not find organicity, we say: well, you probably have irritable bowel syndrome. To diagnose it, you rule out other pathologies; there is no marker that I know of.”*

(General Practitioner, EE04)

Partly, this requirement to go through a set of tests to find nothing so that IBS can emerge creates some discomfort amongst professionals. As this participant emphasized, there is a gap between the imperative (set by currently dominant biomedical paradigms) of needing a positive clinical diagnosis and the reality of having to exclude other conditions to identify IBS:

*“P13: The diagnosis should be clinical, not a diagnosis of exclusion, but the reality is that you have to exclude other pathologies. Currently, there is no test that allows you to tell the patient: look, you have tested positive for irritable bowel syndrome.”*

(Gastroenterologist, EE13)

### 3.3. Pragmatic Diagnostic Strategies

As mentioned above, clinicians often combine multiple sources of information and co-constructed patient narratives in the diagnostic process. Resources such as patient histories, abdominal palpation, bowel rhythm, food intolerance, psychosocial context, and selective use of tests such as blood tests, stool tests, ultrasound, colonoscopy, or other procedures converge in this process.

Interestingly, several participants used the term “despistatge/despistaje” (etymologically rooted in tracking or clearing clues to rule out alternatives) to describe this process. In this context, the term referred to ruling out other possible diseases before considering IBS, rather than population screening, which would be the standard translation and has another word more commonly used (i.e., cribrate). This wording reflected how IBS diagnosis was often experienced as an exclusionary process. However, it is not only a matter of adequate labeling in reality. It is particularly relevant to avoid missing more serious organic conditions and reassure clinicians in follow-up decision-making:

*“P14: I always want to rule out pathology. I try to make sure the patient has previously been assessed by gastroenterology to rule out problems such as organic pathology, malabsorption, or intolerances. Before that, they have usually had analytical tests, parasite tests, or colonoscopies.”*

(Rehabilitation specialist, EE14)

In this sense, diagnostic decision-making was shaped by the pragmatic need to avoid both under-investigation and over-investigation. On the one hand, clinicians sought to reassure patients and prevent unnecessary tests, referrals, or treatments. On the other hand, they remained attentive to symptoms that could indicate organic disease or require specialist assessment. Our participants therefore suggest that IBS management requires a continuous balance between protocol-based safety, clinical judgment, and longitudinal knowledge of the individual patient over time.

*“I personally have the perception that [IBS] is a ‘catch-all category’ […] according to the protocol, you do not have to spend [time] looking for more things if they already meet certain criteria and do not have alarm or warning signs […]. Yes, there are the Rome criteria, but anything can fit in there. […] You can easily label someone as having an irritable bowel when maybe […] they have something else that has not been diagnosed.”*

(Primary care physician, EE08:25)

Thus, the diagnostic process was framed less as the straightforward application of a diagnostic label and more as a pragmatic strategy for excluding other pathologies, ruling out alarm signs, and avoiding missed diagnoses that would require a different clinical course of action. In practice, IBS management requires a balance between protocol-based safety, clinical judgment, and knowledge of the individual patient over time. The expert patient with lived experience of IBS described how this diagnostic pathway evolves from (negative) tests towards progressively integrating psychosocial components that open new possibilities for management:

*“P1: I went to the family doctor and explained my symptoms. I remember they did a stool test, and it came back to normal. Then they asked me questions about stress, diet and possible intolerances, and told me that it was probably irritable bowel syndrome.”*

(Expert patient with lived experience of IBS, EE01)

### 3.4. From Symptom Control to Biopsychosocial and Longitudinal Management

Partly because of how healthcare professionals gradually introduced psychosocial aspects, patients emphasize the role of elements such as stress or food experimentation in IBS management.

The expert patient described IBS as strongly connected to stress and bodily awareness:

*“P1: Nerves have always gone to my stomach. When I am nervous, I notice it physically: my stomach tightens, I feel pain, and I become more aware of everything that creates tension in me. For me, irritable bowel syndrome has a very strong mental component.”*

(Expert patient with lived experience of IBS, EE01)

The same participant also described self-management as an ongoing process of identifying personal triggers:

*“P1: I gradually discovered which food suited me and which did not. For example, cow’s milk made me feel very bad, and I also stopped drinking coffee years ago because it affected me in the same way.”*

(Expert patient with lived experience of IBS, EE01)

These accounts were consistent with professional interpretations of IBS as requiring more than pharmacological symptom control, including diet, stress management, lifestyle advice, and education and, in some cases, psychological support.

*“We also use medication that has been shown to improve this […] but it’s like a suit that’s very much tailor-made for each patient […]. All this must also be done in a way that provides substantial support […] simple guidelines for improving lifestyle, and in some cases, behavioral advice too. […] Often, when you sort out their situation and help them manage their stress, these symptoms improve.”*

(Primary care physician, EE10)

Participants repeatedly connected IBS management with diet, stress, mental health, physical activity, bowel habits, and patient education. This reflected a shift from a narrow biomedical model towards a biopsychosocial model aligned with the gut–brain axis. Rather than treating IBS only as a set of isolated symptoms, professionals described the importance of helping patients understand triggers, recognize patterns, and participate actively in self-management.

### 3.5. Potential of Digital and Non-Pharmacological Support

To evaluate technological and non-pharmacological options accurately, participants’ perceptions were categorized into two distinct operational levels: Non-Pharmacological and Physical Interventions for Symptom Relief (Section 3.5.1) and Digital Monitoring and Pattern Recognition in Clinical Practice (Section 3.5.2).

#### 3.5.1. Non-Pharmacological and Physical Interventions for Symptom Relief

In contrast to general digital monitoring apps, participants held distinct perceptions regarding specific physical and mechanical technologies—such as automated colonic massage devices (e.g., MOWOOT) and targeted physical interventions. While general digital tools were valued for data collection, specific mechanical devices were evaluated for their direct, active therapeutic role in replacing or supplementing pharmacological treatments for constipation and gut motility.

As we follow how our expert patient introduced biopsychosocial elements, we see how non-pharmacological approaches emerge as a series of nodes of care practices, capable of symptom relief, but also as triggers of self-management and significant patient education. In this context, approaches such as heat or abdominal massage were mentioned and even prioritized as potentially useful supportive strategies, even over promising pharmacological solutions:

*“P10: Imagine you could invent a fantastic device or… or there were no… with an unlimited health budget or… what do you think would help you most if you could write a letter to the Three Wise Men, right?*

*For example, that they invented some kind of treatment that was directly effective, right?*

*If you take this pill, you get a colon cleanse and it leaves you… like a baby’s bottom, I think there should be a lot more education on a good food culture and also especially on mental health, rather than finding a pill that’ll sort it all out, because I think if you improve… Yeah, I do not think it is so much about finding a solution…*

*And when you’re not feeling well, even something physical, don’t you get massages or things like that?*

*Yeah, I would do things like, a hot shower. Putting water on my stomach, giving me massages…*

*Well, it’s a way… how can I put it… of, if you like, loving yourself a bit. I mean, in that moment you’re doing it, you’re looking after yourself. And your body ends up appreciating it. I know it sounds a bit daft, but it really isn’t. And with the whole irritable bowel thing, one of the main things that really helps, and I always think about this, is breaking the cycle. When I am nervous, if I calm myself down, it gets better.*

*Easy to say, isn’t it?*

*Easy to say. But in the end… you get to a point where you’re like… it’s not that I want to be calm because I won’t go to the loo […] I have to be calm so I don’t go to the loo. I have to stay calm so I can lead a normal life […], eat whatever I fancy, and […] have energy in the morning. […] I wish they’d find a magic [way] so that people could look after themselves more and […] stop worrying so much.*

*And well, you’ll get people who come in with irritable bowel syndrome, which is completely diverse, but I’m convinced that for a very high percentage […] it’s very much linked to the head […] head and diet too, head and emotions. head and diet too, head and emotions.”*

(Expert patient with lived experience of IBS EE01)

#### 3.5.2. Digital Monitoring and Pattern Recognition in Clinical Practice

When discussing digital health broadly, participants evaluated general software tools—such as digital symptom diaries, mobile tracking applications, and electronic Bristol Stool Scale logs—distinctly from specific mechanical or therapeutic devices. Clinicians viewed these general digital tools not as direct active treatments but valued them for their capacity to organize clinically meaningful information in a way that could be used by professionals and patients during care. Making symptoms, bowel patterns, and treatment response more visible over time was highly praised as long as it truly helped lower professional burden and support (not substitute) patient–professional communication.

Several participants referred to the need for longitudinal follow-up of symptoms, bowel habits, triggers, and treatment response. This was especially relevant in a condition described as fluctuating and difficult to confirm through a single test or consultation. The need was not for more data, but for data that could help identify patterns over time:

*“P5: Patients misinterpret certain bowel movements […], for example, they’ll say, ‘I’m just really bad.’ […] I remember two patients I gave a diary to […]. For [one of them], it was about going to the loo every day […], but I say to him, ‘No […] you’ve taken two days over it, but you’ve done it perfectly.’ So […] it’s really useful for them to be able to monitor exactly how many bowel movements they have and the consistency according to the Bristol stool scale, because otherwise, you end up increasing the treatment based on a feeling they have that isn’t founded on anything.”*

(Gastroenterologist, EE05)

Professionals also referred to the potential usefulness of anticipating worsening or decompensation. This was linked to the possibility of detecting changes in symptoms or bowel patterns before they became clinically more difficult to manage:

*“P4: I don’t know, perhaps the stage the person experiencing it is at—if you could give me any information. The stage… I mean, diarrhea or constipation, right? Like, being able to prevent it or predict it in some way, or… That’s what you sometimes try to do; that’s what people tell me about auscultation, in this case.”*

(Primary care physician, EE04)

Participants also emphasized that any digital-based support would need to provide information that was easy to interpret and did not add workload. Clinician-facing outputs were described as useful only if they could be reviewed rapidly and translated into practical decisions during routine care.

## 4. Discussion

This qualitative study shows that IBS is often understood in clinical practice as a catch-all category (or, as mentioned by the participants, tailor’s drawer), namely a phenomenon that emerges in the process of managing uncertainty about a myriad of symptoms that persist over time. That is, a liminal hotspot in which healthcare professionals and patients struggle to move on in the absence of a test but need to integrate fluctuating symptoms, psychosocial factors, patient narratives, selective diagnostic exclusion, and variable treatment response within short and repeated consultations [27].

These findings should be interpreted considering the substantial clinical and healthcare burden of IBS. IBS is a common disorder with a relapsing-remitting course, relevant effects on quality of life, and important direct and indirect costs for healthcare systems. Previous literature has also highlighted that IBS is particularly challenging in primary care because diagnosis is often delayed, symptoms overlap with other gastrointestinal conditions, and the absence of a specific biomarker creates uncertainty for both clinicians and patients [6,10,11]. Our findings add to this literature by showing how this uncertainty is managed in practice not only through the application of diagnostic criteria but also based on the exclusion of alarm signs that clinicians must avoid, such as prematurely discarding established diagnostic or therapeutic measures that offer symptomatic relief. That is, IBS emerges from a logic of foundation by exclusion that, in some cases, can result in underestimating patient experiences that provide key longitudinal knowledge and inform pragmatic decisions about when to investigate, reassure, refer, or adjust treatment [28].

Our findings extend existing qualitative literature on IBS care by shifting the analytical focus from isolated diagnostic episodes to a continuous, longitudinal process of uncertainty management. Prior studies, such as Crocker et al. [23], highlighted general practitioners’ perceptions of interpersonal relationships and diagnostic frustration when dealing with IBS. In contrast, our study demonstrates that clinicians across primary and specialized care do not merely experience uncertainty as a barrier; rather, they actively manage it through pragmatic pattern recognition, long-term patient history, and relational knowledge over time. By identifying IBS management as a “liminal hotspot” operating through a logic of diagnostic exclusion, this research provides new empirical insight into how structured longitudinal tracking and low-burden digital tools can transform persistent clinical ambiguity into actionable, patient-centered care trajectories.

**Figure 1:**
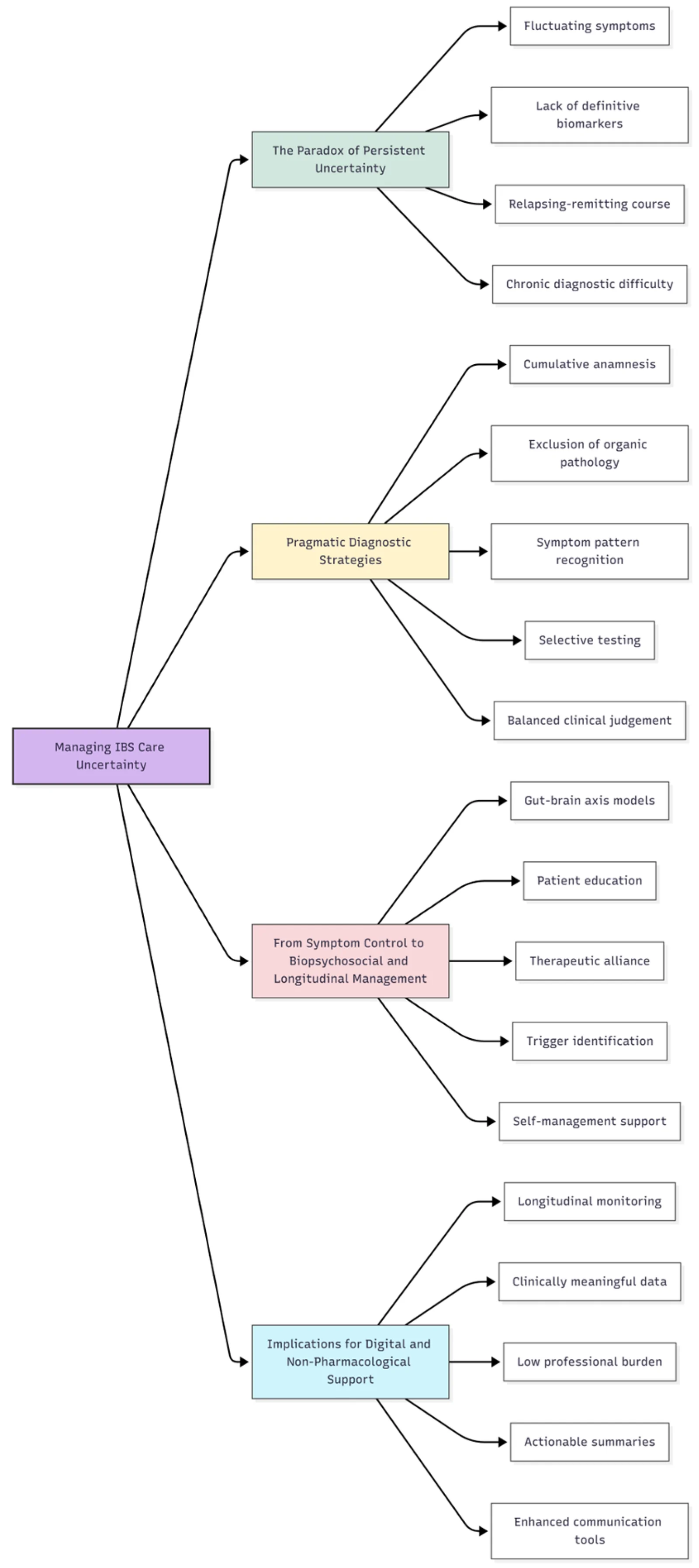
Thematic map of qualitative findings.

Beyond identifying these essential aspects of IBS care and its characteristic of continuous uncertainty, our qualitative findings can be interpreted through the systemic paradigm shift described by Hood [29], who posited that contemporary healthcare is transitioning toward a P4 (Predictive, Personalized, Preventive, Participatory) model. While clinicians in our study did not explicitly use formal theoretical frameworks or articulate their routine in these terms, their emphasis on longitudinal pattern recognition suggests that digital and non-pharmacological tools (such as MOWOOT) can practically align with this P4 framework (Figure 1).

In the context of IBS care, digital tools and non-pharmacological medical technologies (such as MOWOOT) emerge as potential catalysts of these four pillars: they offer personalized data tracking, facilitate predictive recognition of symptom decompensation, support preventive lifestyle strategies, and foster participatory engagement between patients and healthcare providers [29].

The findings are consistent with literature describing IBS as a multidimensional disorder of gut–brain interaction and with clinical reviews emphasizing the burden, unmet needs, and complexity of IBS management [1,2,3,4,5,7,10,16,17]. They also add a practice-oriented perspective: clinicians seem to develop confidence and pragmatic explanations through longitudinal pattern recognition and relational knowledge of the patient [30]. This has implications for any intervention intended for primary care. A useful tool should support this longitudinal reasoning rather than replace it with isolated measurements.

The relevance of context was also evident when comparing our findings with recent evidence on IBS management in specialist practice. A recent survey of Latin American gastroenterologists showed broad use of Rome IV criteria but also substantial variability in diagnostic testing and treatment choices, influenced by local availability, clinical practice patterns, and healthcare system factors [31]. This supports the interpretation that IBS management is not only a question of applying guidelines but also of adapting decisions to real-world constraints, available resources and explanations, and professional workflows [32].

The study also supports the relevance of digital health in IBS under specific conditions. Across the analytical themes, technology adoption is shaped by the need for clinically interpretable data and low professional burden; structured patient-reported information that identifies patterns without causing fatigue; and, additionally, it relies on tools that reinforce the therapeutic alliance rather than replacing clinical judgment. Ultimately, rather than functioning as stand-alone technologies, digital and non-pharmacological support tools should help organize longitudinal symptom trajectories and self-management needs into brief, actionable summaries. These tools may effectively complement clinical workflows, serving as key design implications summarized in Table 2.

This study contributes to the literature by showing that, in primary care, IBS uncertainty is not only a diagnostic problem related to the absence of biomarkers but also an organizational and relational process. Professionals manage IBS through repeated encounters, knowledge of the patient, selective exclusion of alarm features, and negotiation of symptom meaning. This perspective is particularly relevant for the design of support tools, which should complement rather than replace clinical judgment.

Table 2 summarizes the main implications derived from the four analytical themes for IBS care and digital or non-pharmacological support.

### 4.1. Strengths and Limitations

A strength of the study is the inclusion of different professional profiles and the methodological guidance provided by one expert patient with lived experience of IBS, allowing comparison and articulation across care levels and perspectives. The qualitative design was appropriate for exploring clinical reasoning, implementation of barriers, and patient–professional interaction. However, the findings should be interpreted considering several limitations. The sample was purposive and located in one metropolitan healthcare context; therefore, transferability to other settings should be assessed cautiously. Although the study included a diverse range of professional profiles, the number of participants within each profile was limited, and the patient’s perspective was represented by only one expert patient with lived experience of IBS. No formal statistical sample size calculation was conducted, as the study followed a qualitative, purposive sampling strategy. The analysis was conducted in the context of a technology-development project, which may have oriented attention towards data, monitoring, and usability.

### 4.2. Implications for Future Research

Long-Term Patient Studies: Future research should track patients over longer periods to see how using digital symptom logs or device treatments affects how often they visit the doctor and how their symptoms change over time.
Testing in Different Clinics and Teams: Studies should compare how these tools work in different settings—such as public primary care centers versus private clinics—and include other healthcare workers like nurses, dietitians, and psychologists.
Real-World Clinical Testing: Researchers need to test physical devices (like colonic massage tools) and apps in everyday clinic visits to measure whether they save time for doctors and genuinely help patients manage their care.

## 5. Conclusions

According to the healthcare professionals interviewed, IBS management in this primary care context operates as a longitudinal process of diagnostic and therapeutic uncertainty management. Professionals combine exclusion of alternative diagnoses, symptom interpretation, patient narrative, therapeutic trial-and-error, and psychosocial understanding to support care. Digital and non-pharmacological interventions may be valuable when they help organize this uncertainty, support patient self-management, and provide interpretable information that fits primary care workflow. These findings may inform the development of digital and non-pharmacological support tools, including MOWOOT-IBS, by emphasizing the importance of monitoring, education, and shared management in IBS care.

## Figures and Tables

**Table 1:** Participant profiles are represented in the qualitative dataset.

Participant Profile	n	Reason for Inclusion	Care Level/Setting	Years of Experience (Range)
Expert patient with lived experience of IBS	1	Exploratory/pilot input to guide methodological choices and patient care pathways	Community/Lived Experience	N/A
Family and community medicine/primary care physicians	6	To capture the setting where much IBS diagnosis and longitudinal management occurs.	Primary Care	5–25 years
Nursing professionals	3	To explore education, follow-up, and self-management support.	Primary Care	6–18 years
Gastroenterology specialists	3	To contrast specialist diagnostic and therapeutic perspectives.	Specialized/Hospital Care	8–20 years
Medical resident	1	To incorporate an early-career clinical perspective on uncertainty.	Primary/Hospital Rotations	2 years
Rehabilitation/pelvic floor specialist	1	To include functional, physical, and non-pharmacological approaches relevant to bowel symptoms.	Specialized Care	12 years

**Table 2:** Analytical themes and implications for IBS care and digital/non-pharmacological support.

Theme	Core Finding	Implication for Primary Care or Digital Support
The Paradox of Persistent Uncertainty	IBS was described as difficult to confirm, stabilize, or explain because of fluctuating symptoms and lack of definitive biomarkers.	Tools are suggested to focus on organizing uncertainty and detecting changes over time, rather than promising diagnostic certainty.
Pragmatic diagnostic strategies	Clinicians combined anamnesis, abdominal examination, tests, exclusion of other pathology, and symptom patterns.	Structured clinical histories and longitudinal data could assist clinicians in supporting complex decision-making.
From Symptom Control to Biopsychosocial and Longitudinal Management	Diet, stress, mental health, bowel habits, patient education, and therapeutic alliance were central to care.	Digital health applications may better serve clinical workflows by facilitating self-management and patient–provider communication, rather than solely recording biomedical variables.
Implications for Digital and Non-Pharmacological Support	Time pressure, workload, and the need for clinically meaningful information shaped technology adoption.	Interfaces could prioritize simple, low-burden designs oriented toward providing actionable summaries for clinicians.

## Data Availability

The qualitative datasets generated and analyzed during the current study are not publicly available because they contain interview transcripts that could compromise participant confidentiality. Anonymized excerpts supporting the findings are included in the manuscript.

## Supplementary Materials

The following supporting information can be downloaded at: https://www.mdpi.com/article/10.3390/healthcare14182928/s1, File S1: Interview Script (basic script that may vary in the different phases and according to the type of interview).

## Abbreviations

The following abbreviations are used in this manuscript:

DGBI	Disorder of gut–brain interaction
IBS	Irritable bowel syndrome
IBS-C	Constipation-predominant irritable bowel syndrome
