# Supplementary material for: Irritable Bowel Syndrome Care in Catalonia: A Qualitative Study with Healthcare Professionals on the Management of Uncertainty and the Possibilities of Technological and Digital Developments
Source: Healthcare (Basel). 2026 Sep 9;14(18):2928. doi: 10.3390/healthcare14182928 (PMC13606812; doi:10.3390/healthcare14182928)
Supplement: Supplementary file 1 [file healthcare-14-02928-s001.zip › healthcare-4540247-supplementary.pdf]

Intreview Script ***(basic script that may vary in the different phases and according to the type of interview).***

PRESENTATION

- ✓ **Thank them** for their cooperation.
- ✓ Identify ourselves: **who we are**, which organisation backs us, who is sponsoring the research, what it involves…
- ✓ Specify the thematic **purpose** of the interview: why we are conducting it, what will be discussed, and our interest in their opinion and experience.
- ✓ Explain the interview's **format**: an informal conversation, a relaxed dialogue, questions and answers, duration, etc.
- ✓ Ask for **permission** to record the audio, **ensuring anonymity and confidentiality**, and explicitly ask if there are **any doubts.**
- ✓ Emphasise that participation is voluntary!

- **Introduction of the interviewers.**

I am Feu click here to write text., Feu click here to write text..

First of all, thank you enormously for agreeing to take part in this interview.

With this information, we will be able to identify the main needs, especially regarding the collection of information, for the diagnosis and management of irritable bowel syndrome.

I want to reiterate that your participation must be entirely voluntary. Therefore, in addition to requesting your written **consent**, I remind you that you may withdraw from participation at any time and without having to give any explanation.

Furthermore, I would like to remind you that **we will be recording the audio** to retain a record of what you say and to analyse the content in depth afterwards. However, I can assure you that your data and opinions will be treated with the utmost confidentiality (the recordings and transcripts will be stored on ICS' server, with limited access for the research team). Also, should any extract be made public, it will be done in such a way that at no point will it be possible to identify you.

Finally, if you wish, we could provide you with a **feedback** on the research results at its conclusion. To this end, if you are interested, please indicate a contact email address on the consent form.

- **Session methodology:**

We are particularly interested in your experience and your opinions. For this reason, you should not feel inhibited from expressing your point of view; on the contrary, that is exactly what we are looking for. There are no right or wrong answers to the questions we will all be asking. We have a number of topics we wish to discuss, but we will introduce them as the conversation unfolds.

The meeting **will last no more than 60 minutes.** However, you can step away temporarily or leave permanently whenever you wish. We will start the recording straight away.

DEVELOPMENT[1]

- ✓ THEMATIC GUIDE, not a prescription→ indicative guide to the contents to be covered
- ✓ The sequence and questions can be altered by
  - ✓ Follow the conversational thread proposed by the interviewee
  - ✓ Delve into EMERGING topics
- ✓ Ask for explanations, examples and clarifications of any aspects that are not clear at the time the interviewee mentions them.
- ✓ Minimise the use of yes/no questions (better: what, how, what, when, why…).
- ✓ Make original suggestions (scenarios, situations, ask for stories, etc.)

Introductory question (can provide a different, non-sequential and more spontaneous entry into the thematic blocks):

If you'd like, please introduce yourself briefly.

What is the first thing that comes to mind when you hear about irritable bowel/colon?

TOPIC BLOCKS: (from the general to the most specific)

A. **Diagnosis and management**
   a. How is irritable bowel syndrome suspected? (timing, key words, etc.):
      i. Key elements of the patient's history
      ii. Physical examination (circumference, sounds, rhythms, etc.)
      iii. Symptomatology
   b. What steps do you take in this situation?
   c. Which tools are the most important in this process?
   d. Which professionals are or should be involved in the diagnosis and treatment of CI?
   e. What role do you think the patient plays at this point?
   f. How do you record the data for this patient and their examination?
   g. What barriers and facilitators do you encounter in the diagnostic process?

B. Profiles
   a. How would you describe a "typical" patient with an irritable bowel/colon (IBS) diagnosis?
   b. Can you describe any case that gives you doubts (either due to lack of information, non-specific symptoms, etc.)?
   c. How would you characterise the experience of this condition?

C. Key **needs and information**:
   a. What are the key questions to ask when suspecting IBS?

[1] A semi-structured interview proposal is presented; it should be adapted to the context and modality, if appropriate.

    b. Which tests help you identify this condition?
    c. What indicators help you refine the diagnosis?
    d. What else would you like to know if you could gather more data on that patient?

D. <u>"Letter to the Wise Men":</u>
    a. If you could invent a device to identify this pathology, what parameters would it collect?
        i. Assess the intensity, frequency, etc. of the recordings.
        ii. Suggest some if appropriate.
    b. How do you imagine an ideal doctor-patient consultation/relationship for CI?

CLOSURE

- ✓ Ensure that all topics on the script have been adequately covered.
- ✓ Give the interviewee the opportunity to add, modify or clarify any aspect of their narrative
- ✓ Thanking the informant for their collaboration
- ✓ Securing a commitment to return the evaluation results (by sending the evaluation report or indicating where it can be found)

Would you like to add any further questions? If not, this session concludes here.

Thank you very much for coming and sharing your time and experiences; they will be very useful for advancing our research.
